# Unilateral Pedicle Stress Fracture in a Long-Term Hemodialysis Patient with Isthmic Spondylolisthesis

**DOI:** 10.1155/2015/426940

**Published:** 2015-02-08

**Authors:** Keishi Maruo, Toshiya Tachibana, Shinichi Inoue, Fumihiro Arizumi, Shinichi Yoshiya

**Affiliations:** Department of Orthopaedic Surgery, Hyogo College of Medicine, Mukogawa-cho 1-1, Nishinomiya, Hyogo 663-8501, Japan

## Abstract

Most unilateral pedicle stress fractures occur on the contralateral side of patients with unilateral spondylolysis. However, there are few reports of unilateral pedicle stress fractures in patients with bilateral spondylolysis and spondylolisthesis. We report a unique case of unilateral pedicle stress fracture in a long-term hemodialysis patient with isthmic spondylolisthesis. A 65-year-old man who had undergone hemodialysis presented with lower back pain that had persisted for several years. The patient experienced severe right lower extremity pain with no history of trauma. Computed tomography revealed unilateral pedicle fracture with bilateral L5 spondylolysis and spondylolisthesis with progression of scoliosis. The patient underwent Gill laminectomy of L5 with pedicle screw fixation at L4-S1 and interbody fusion at L5-S1. The patient's leg pain ceased immediately, and he began walking without leg pain. In our present patient, development of scoliosis caused by destructive spondyloarthropathy may have contributed to a unilateral pedicle fracture.

## 1. Introduction

Stress fractures of the pedicle are uncommon. There are reports of bilateral pedicle stress fractures in patients with osteoporotic conditions [1–3] or in association with previous spinal surgery [4–7] as well as reports of unilateral pedicle stress fractures associated with contralateral spondylolysis [8–11]. However, there are few reports of unilateral pedicle stress fractures in patients with bilateral spondylolysis. We report here an uncommon case of a patient with unilateral pedicle stress fracture of the L5 vertebra with no history of trauma. The patient was previously treated with long-term hemodialysis (18 years), and he had isthmic spondylolisthesis.

## 2. Case Report

**Figure 1:**
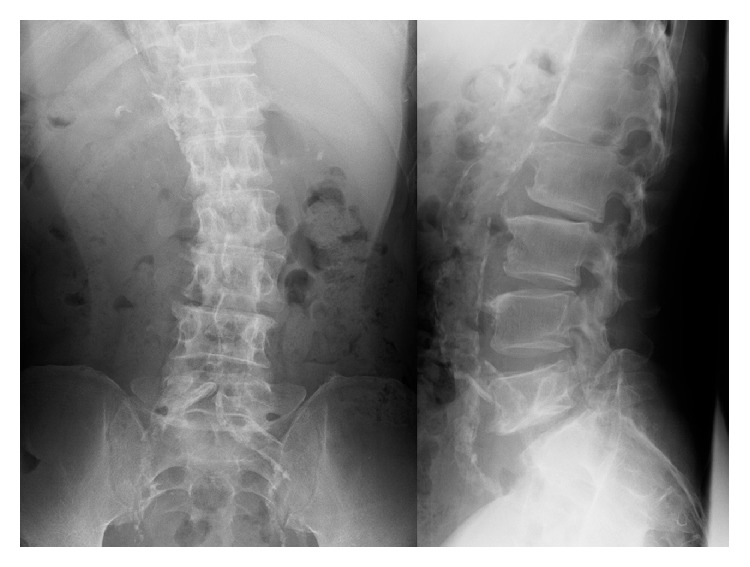
Plain radiographs showing a degenerative lumbar scoliosis and Meyerding grade I L5-S1 isthmic spondylolisthesis 2 years before surgery.

**Figure 2:**
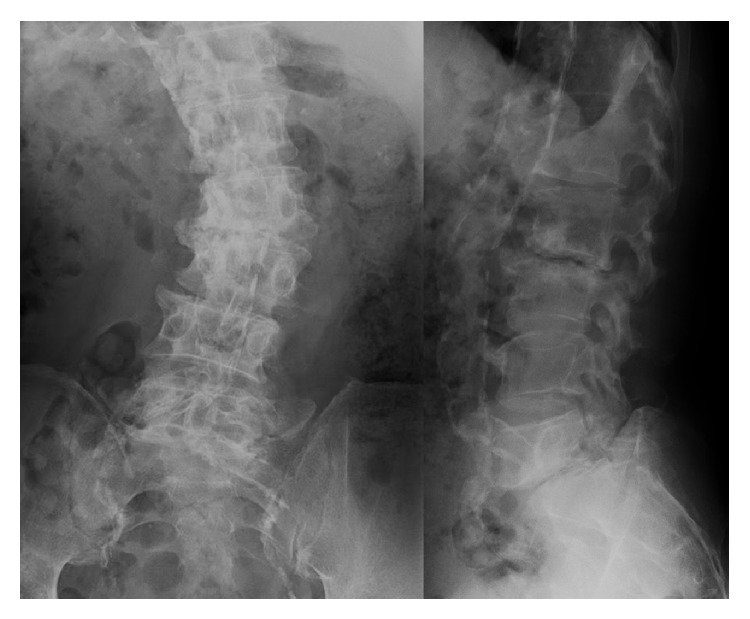
Preoperative plain radiographs showing progression of scoliosis and destructive changes at L2-L3 and L5-S1.

**Figure 3:**
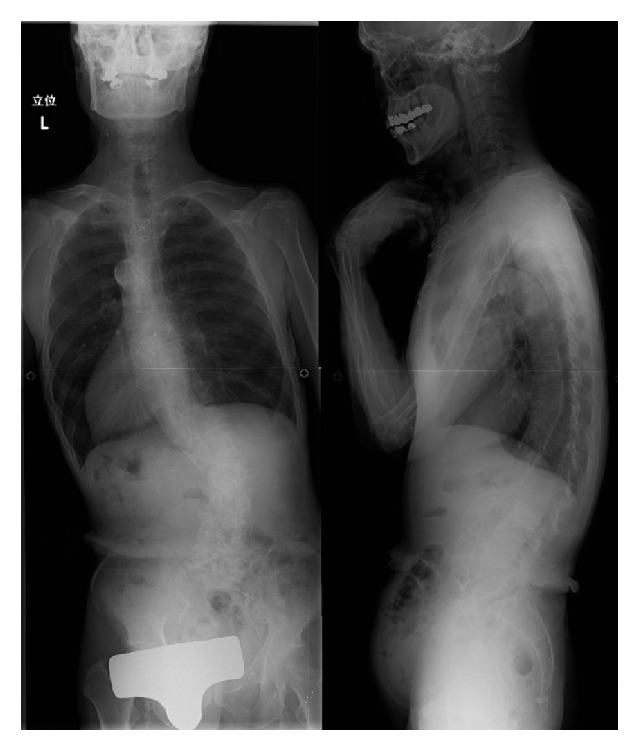
Preoperative radiographs showing right convex lumbar curvature and left-side coronal imbalance.

**Figure 4:**
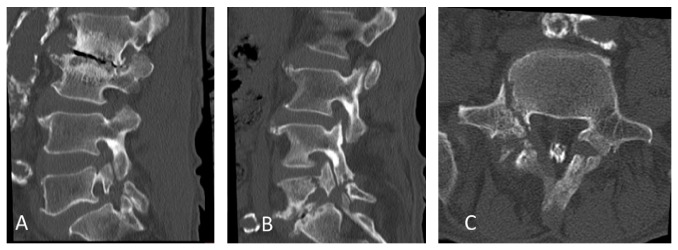
Sagittal reconstruction CT confirming bilateral spondylolysis and destructive changes at L2-L3 and L5-S1 (A, B). Axial CT and right-side parasagittal reconstruction CT confirming right-side L5 pedicle fractures (B, C).

**Figure 5:**
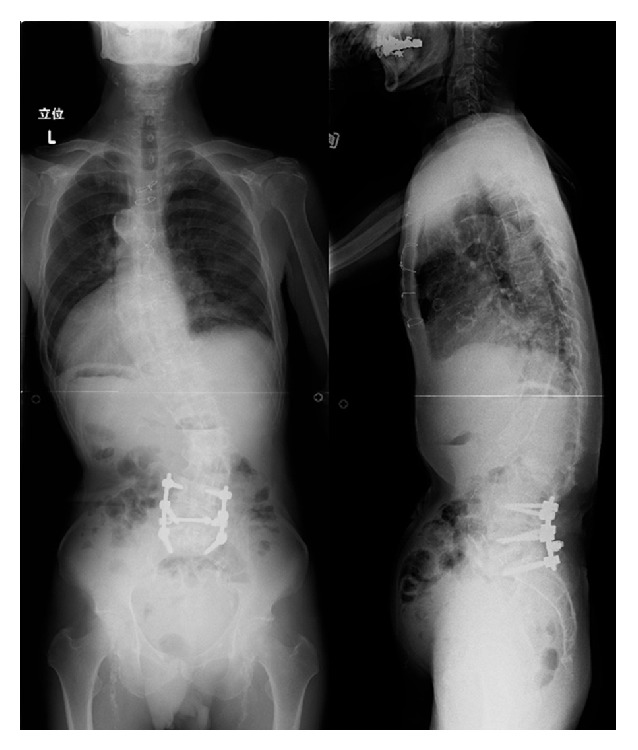
Postoperative radiographs showing coronal imbalance and pelvic retroversion that improved after surgery.

A 65-year-old man presented with lower back pain that had persisted for several years. The patient experienced severe right lower extremity pain for several weeks with no history of trauma. He could only walk fewer than 10 m due to severe lower extremity pain. He had no history of spinal surgery. The patient was treated at a local hospital with analgesics that were administered using an epidural block. However, intermittent claudication and pain progressed due to right lower extremity pain. Motor examination revealed grade 4 muscle weakness in the tibialis anterior and the extensor hallucis longus as well as hypesthesia in the L5 area. Plain radiographs acquired in 2010 revealed degenerative lumbar scoliosis and Meyerding grade I isthmic spondylolisthesis at L5-S1. There was no evidence of destructive changes such as those associated with long-term hemodialysis (Figure 1). In 2012, destructive spondyloarthropathy (DSA) was detected using plain radiographs and manifested as erosion of the vertebral endplate and disc-space narrowing at L2-L3 and L5-S1. This caused the progression of lumbar curvature from 18° to 35°, and anterior slippage of L5 increased from 8% to 15% at L5-S1 (Figure 2). Global spinal alignment demonstrated left-side coronal imbalance to 101 mm and pelvic retroversion to 26° (Figure 3). Computed tomography (CT) performed after myelography revealed a right side L5 pedicle fracture, bilateral L5 spondylolysis, L5-S1 spondylolisthesis, and erosive changes of the vertebral endplate at L5-S1 (Figure 4). Surgery was considered due to persistent, severe leg pain. The patient had a history of aortic valve replacement for ischemic heart disease. In addition, he was receiving antiplatelet therapy. Limited decompression and fusion surgery was selected despite sagittal global malalignment because his symptom localized to the L5 region and we considered the patient's comorbidities. The patient underwent Gill laminectomy of L5 with pedicle screw fixation at L4-S1 and interbody fusion using the posterior lumbar interbody fusion (PLIF) technique at L5-S1. A thoracolumbar spinal orthosis brace was used for 3 months after surgery. The patient's leg pain ceased immediately, and he began walking three days later with normalization of his examination. Postoperative radiographs at the one-year follow-up showed improvement of coronal imbalance from 101 mm to 38 mm and loosening of left L4 pedicle screw. Postoperative pelvic retroversion decreased from 26° to 17° (Figure 5), and postoperative sagittal vertical axis remained greater than 50 mm (52 mm) due to thoracolumbar kyphosis (17°). The patient was without right lower extremity pain but was experiencing residual low back pain due to pseudoarthrosis at the one-year follow-up.

## 3. Discussion

Stress fractures of the pedicle are much less common than those of the pars interarticularis. Most of the reported pedicle stress fracture cases occurred bilaterally [1–3] or on the contralateral side with unilateral spondylolysis [8–10]. Patients with bilateral pedicle stress fractures often had bone fragility, including rheumatoid arthritis [2], osteoporosis [1], and ankylosing spondylitis (AS) [3]. Previous spinal surgery also causes bilateral pedicle fracture [4–7]. Unilateral pedicle stress fractures associated with contralateral spondylolysis occur in patients engaged in competitive sports [8, 10]. Our present patient suffered from isthmic spondylolisthesis with no history of trauma. To our knowledge, there is only one other report of isolated unilateral pedicle fracture in a patient with bilateral spondylolysis [12], which was caused by minor trauma including a back massage. However, there are no published reports of unilateral pedicle stress fracture in a patient with bilateral spondylolysis and spondylolisthesis with no history of trauma.

The mechanism responsible for the unilateral pedicle fracture is unknown. Biomechanical studies show that the pedicle is the second weakest portion of the neural arch [13]. El-Rich et al. demonstrated a high level of equivalent stress in the pars interarticularis and pedicle of L5 in low-grade isthmic spondylolisthesis using a personalized finite element model [14]. In the present case, bending stress caused by progression of scoliosis may have influenced the onset of the unilateral pedicle fracture at the concavity of L4-L5. Furthermore, the destructive change of L2-L3 due to amyloidosis led to progression of L2-L3 scoliosis. A destructive change involving L5-S1 also contributed to the progression of L5 slippage, which caused the pedicle fracture due to increased unilateral bending forces.

Surgical options for pedicle fracture include screw placement through the pedicle into the vertebral body [15], posterolateral fusion (PLF) [16], PLIF, or transforaminal lumbar interbody fusion. In the present case, we decided to perform a PLIF at L5-S1 with Gill laminectomy, and PLF was performed at L4-L5 to prevent progression of DSA. Long fusion such as lower thoracic spine extending to the ilium may lead to better long-term results for patients with adult spinal deformity. We recommend safer and less-invasive surgery for patients undergoing long-term hemodialysis. Our present patient's pain was completely alleviated, and he enjoyed a full return to normal function at the time of his short-term follow-up.

There are some limitations of our study. First, we lacked bone mineral density data. Whether an association exists between bone mineral density and fracture in hemodialysis patients is unknown [17]. Furthermore, patients with dialysis often suffer from underlying metabolic bone diseases, which may be associated with an increased risk of fractures. Second, the postoperative follow-up period may not have been sufficient to assess the clinical outcome.

In conclusion, unilateral pedicle stress fracture in patients with isthmic spondylolisthesis is rare. The development of scoliosis caused by DSA may have influenced the occurrence of unilateral pedicle fracture in our patient described. Limited decompression and fusion surgery led to a successful outcome.

## References

[1] Doita M., Ando Y., Hirata S., Ishikawa H., Kurosaka M. (2009). Bilateral pedicle stress fracture in a patient with osteoporotic compression fracture. *European Spine Journal*.

[2] Hajjioui A., Khazzani H., Sbihi S., Bahiri R., Benchekroune B., Hajjaj-Hassouni N. (2011). Spondylolisthesis on bilateral pedicle stress fracture in the lumbar spine: a case study. *Annals of Physical and Rehabilitation Medicine*.

[3] Kim H. S., Ju C. I., Kim S. W. (2010). Bilateral pedicle stress fracture accompanying spondylolysis in a patient with ankylosing spondylitis. *Journal of Korean Neurosurgical Society*.

[4] Ha K.-Y., Kim Y.-H. (2003). Bilateral pedicle stress fracture after instrumented posterolateral lumbar fusion: a case report. *Spine*.

[5] Sheehan J. P., Helm G. A., Sheehan J. M., Jane J. A. (2002). Stress fracture of the pedicle after extensive decompression and contralateral posterior fusion for lumbar stenosis. Report of three cases. *Neurosurgical Focus*.

[6] Macdessi S. J., Leong A. K., Bentivoglio J. E. C. (2001). Pedicle fracture after instrumented posterolateral lumbar fusion: a case report. *Spine*.

[7] Robertson P. A., Grobler L. J. (1993). Stress fracture of the pedicle: a late complication of posterolateral lumbar fusion. *Spine*.

[8] Jeong I.-H., Hwang E.-H., Bae W.-T. (2009). Contralateral pedicular fracture with unilateral spondylolysis. *Journal of Korean Neurosurgical Society*.

[9] Gunzburg R., Fraser R. D. (1991). Stress fracture of the lumbar pedicle: case reports of ‘pediculolysis’ and review of the literature. *Spine*.

[10] Sairyo K., Katoh S., Sasa T. (2005). Athletes with unilateral spondylolysis are at risk of stress fracture at the contralateral pedicle and pars interarticularis: a clinical and biomechanical study. *The American Journal of Sports Medicine*.

[11] Vialle R., Mary P., de Carvalho A., Pointe H. D. L., Damsin J.-P., Filipe G. (2007). Acute L5 pedicle fracture and contralateral spondylolysis in a 12-year-old boy: a case report. *European Spine Journal*.

[12] Guo Z., Chen W., Su Y., Yuan J., Zhang Y. (2013). solated unilateral vertebral pedicle fracture caused by a back massage in an elderly patient: a case report and literature review. *European Journal of Orthopaedic Surgery & Traumatology*.

[13] Cyron B. M., Hutton W. C. (1978). The fatigue strength of the lumbar neural arch in spondylolysis. *The Journal of Bone and Joint Surgery—British Volume*.

[14] El-Rich M., Aubin C. E., Villemure I., Labelle H. (2006). A biomechanical study of L5-S1 low-grade isthmic spondylolisthesis using a personalized finite element model. *Studies in Health Technology and Informatics*.

[15] Johnson J. N., Wang M. Y. (2009). Stress fracture of the lumbar pedicle bilaterally: Surgical repair using a percutaneous minimally invasive technique–case report. *Journal of Neurosurgery: Spine*.

[16] Doita M., Shimomura T., Nishida K., Maeno K., Fujioka H., Kurosaka M. (2008). Bilateral pedicle stress fracture in a patient with lumbar spinal stenosis: a case report. *Journal of Spinal Disorders & Techniques*.

[17] Jamal S. A., Hayden J. A., Beyene J. (2007). Low bone mineral density and fractures in long-term hemodialysis patients: a meta-analysis. *The American Journal of Kidney Diseases*.

